# Selective toxicity of tumor treating fields to melanoma: an in vitro and in vivo study

**DOI:** 10.1038/s41420-018-0106-x

**Published:** 2018-10-03

**Authors:** Yunhui Jo, Sang-Gu Hwang, Yeung Bae Jin, Jiwon Sung, Youn Kyoung Jeong, Jeong Hwa Baek, Jae-Min Cho, Eun Ho Kim, Myonggeun Yoon

**Affiliations:** 1 Division of Radiation Biomedical Research, Korea Institute of Radiological and Medical Sciences, Seoul, 01812 Republic of Korea; 2 Department of Bio-Convergence Engineering, Korea University, Seoul, 02842 Republic of Korea; 3 National Primate Research Center, Korea Research Institute of Bioscience and Biotechnology, Daejeon, 34141 Republic of Korea; 4 Radiation Non-Clinical Center, Korea Institute of Radiological and Medical Sciences, Seoul, 01812 Republic of Korea

**Keywords:** Cancer, Cancer

## Abstract

Tumor treating fields (TTFs) are a newly developed cancer therapy technology using an alternating electric field that may be a possible candidate for overcoming the limitations of conventional treatment methods currently used in cancer treatment. Although clinical results using TTFs appear promising, concerns regarding side effects must be clarified to demonstrate the effectiveness of this treatment method. To investigate the side effects of TTF treatment, the damage to normal cell lines and normal tissue of a mouse model was compared with the damage to tumor cells and tumors in a mouse model after TTF treatment. No serious damage was found in the normal cells and normal tissues of the mouse model, suggesting that the side effects of TTF treatment may not be serious. Our evidence based on in vitro and in vivo experiments suggests that TTF may cause selective damage to cancer cells, further demonstrating the potential of TTF as an attractive alternative to conventional cancer treatment modalities.

## Introduction

Despite desperate efforts, cancer is among the most urgent public health problems worldwide, accounting for 1 of 6 deaths. While progress is continuously achieved in conventional treatment modalities, such as surgery, radiation therapy, and chemotherapy, these treatment modalities still have some limitations and difficulties in treating certain cases of cancer.

Recently, a new cancer treatment technique (called tumor treating fields (TTFs)) using ‘alternating electric fields’ has been reported to result in an excellent therapeutic effect on glioblastoma multiform (GBM), which is among the refractory cancers treated using the aforementioned conventional therapies^1^. A randomized phase III trial treating patients with newly diagnosed glioblastomas with temozolomide (TMZ) alone or a combination of TMZ and TTFs showed that most clinical results, such as the median overall survival, progression-free survival, and longer-term survival, were superior with the combined TMZ and TTF treatment compared with those with TMZ monotherapy^2^. Thus, TTFs were recommended as a standard treatment for patients with GBM by the National Comprehensive Cancer Network (NCCN)^3^ and acquired the US Food and Drug Administration (FDA) approval in the United States and CE mark in Europe^4^.

Previous studies suggested that TTFs, which involve an alternating electric field of low intensity and intermediate frequency, can suppress mitosis by interfering with the alignment of the spindle and lead to cell cycle arrest at the G2/M phase and cell death^1,5^. TTFs have been reported to selectively act on fast growing cells rather than slow growing cells, suggesting that TTFs cause more significant damage to cancer cells than to slow growing normal cells. To date, clinical results have indicated that one of the most frequent side effects in patients treated with TTFs is local skin irritation mainly due to the need to attach electrodes to the skin around the tumor^6^. Kirson et al.^1^ also reported that the mesenchymal and diaphragm viable cell numbers in rats treated with TTFs under the conditions of 1.2 V/cm intensity and 100 kHz frequency for 24 days did not differ from those in the control group. Although clinical results suggest that the side effects experienced by treated patients are reported as less severe than those following conventional cancer therapies^2,7^, there is concern regarding normal tissue damage following TTFs resulting in side effects and expanding the clinical application of TTFs; thus, experimentally clarifying the adverse effects of TTF therapy based on in vitro and in vivo experiments is essential.

To clarify the side effects of TTF treatment, we investigated the damage to normal cell lines and normal tissue in a mouse model after TTF treatment. In the in vivo experiments, melanoma cells were injected, and TTF treatment was applied, resulting in therapeutic effects on the subcutaneously injected melanoma cells in the mice^8^. In the in vivo studies, normal tissue from organs in a mouse model were collected after TTF treatment and tested using hematoxylin and eosin (H&E) staining and terminal deoxynucleotidyl transferase-mediated dUTP nick end labeling (TUNEL) assays. In the in vitro experiments, to determine whether the results are consistent with patient samples, we tested the response to TTF applications in malignant tumors and normal cells derived from the *same* patient. In this study, the details of TTF-induced damage to normal cell lines and normal tissue in a mouse model are shown and discussed by comparing this damage to TTF-induced damage to tumor cell lines and tumor tissue in a mouse model.

## Results

### TTF treatment inhibits proliferation and induces cell death selectively in cancer cells but not in normal cells in vitro

**Fig. 1:**
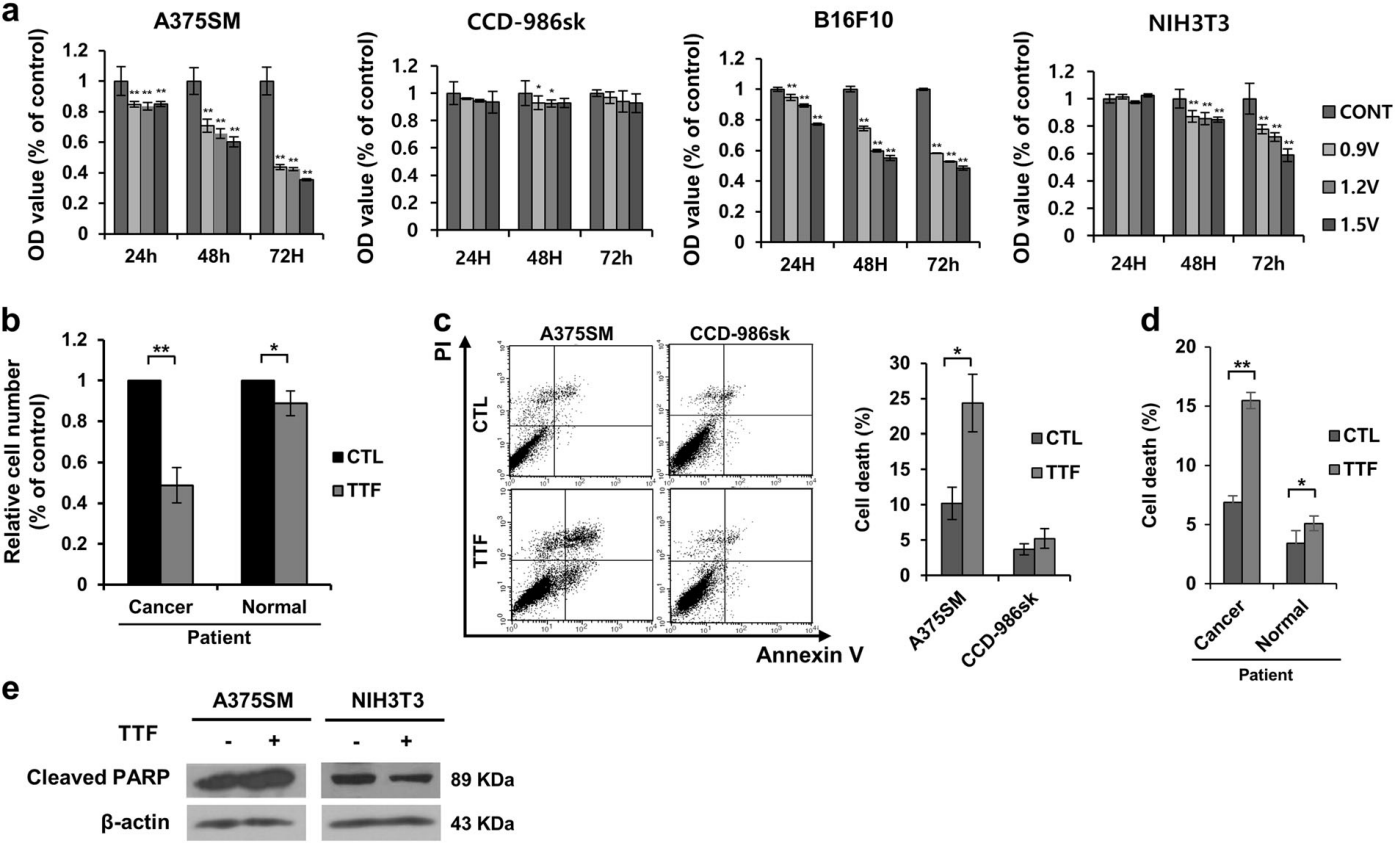
TTFs selectively inhibit tumor cell growth and induce cell death. **a** Tumor (A275SM and B16F10) and normal (CCD-986sk and NIH3T3) cells were treated with TTFs for 48 h and immediately harvested. Cell viability assays of the tumor and normal cells were performed in a 96-well culture dish. **b** Patient-derived tumor and normal cells were treated with TTFs for 48 h. Cell viability assays were performed in a 96-well culture dish. **c**, **d** Cell death rates in the cancer and normal cells were assessed by flow cytometry. **e** Equal amounts of cell lysates (20 µg) were separated by electrophoresis and analyzed by western blotting using the indicated antibodies. The values represent the means ± SD; *p < 0.05, **p < 0.01

TTFs have been reported to inhibit proliferation in brain cancer cells^9^. We examined the inhibitory effect of TTF treatment on cancer and normal cell proliferation using malignant melanoma cells. TTFs were applied to A375SM (human melanoma cells), CCD-986sk (human skin normal cells), B16F10 (mouse melanoma cells), and NIH3T3 (mouse embryo cells) cells for 48 h, and the cells were immediately harvested. The TTF treatment inhibited proliferation in the cancer cells to a greater extent than that in the normal cells (Fig. 1a). In addition, the same tendency was observed when the experiment was performed using cancer cells and normal cells derived from patients (Fig. 1b).

To determine whether this inhibition of proliferation is due to cell death, Annexin V-fluorescein isothiocyanate (FITC)/propidium iodide (PI) staining was performed in melanoma cells and normal cells by referring to a report stating that TTFs induce apoptosis in cancer cells^10–13^. The flow cytometry analysis showed that the rate of cell death after the TTF treatment increased in the cancer cells but not in the normal cells (Fig. 1c). The same tendency was observed when the experiment was performed using cancer cells and normal cells derived from patients (Fig. 1d). In addition, increased cleaved poly (ADP-ribose) polymerase (PARP) was selectively detected in the cancer cells (Fig. 1e). Therefore, cell death was almost induced in a nearly insignificant fashion by TTF treatment in the normal cells, while a significant increase was observed in cancer cells^10^.

### TTFs induce double-strand breaks (DSBs) only in cancer cells but not in normal cells

**Fig. 2:**
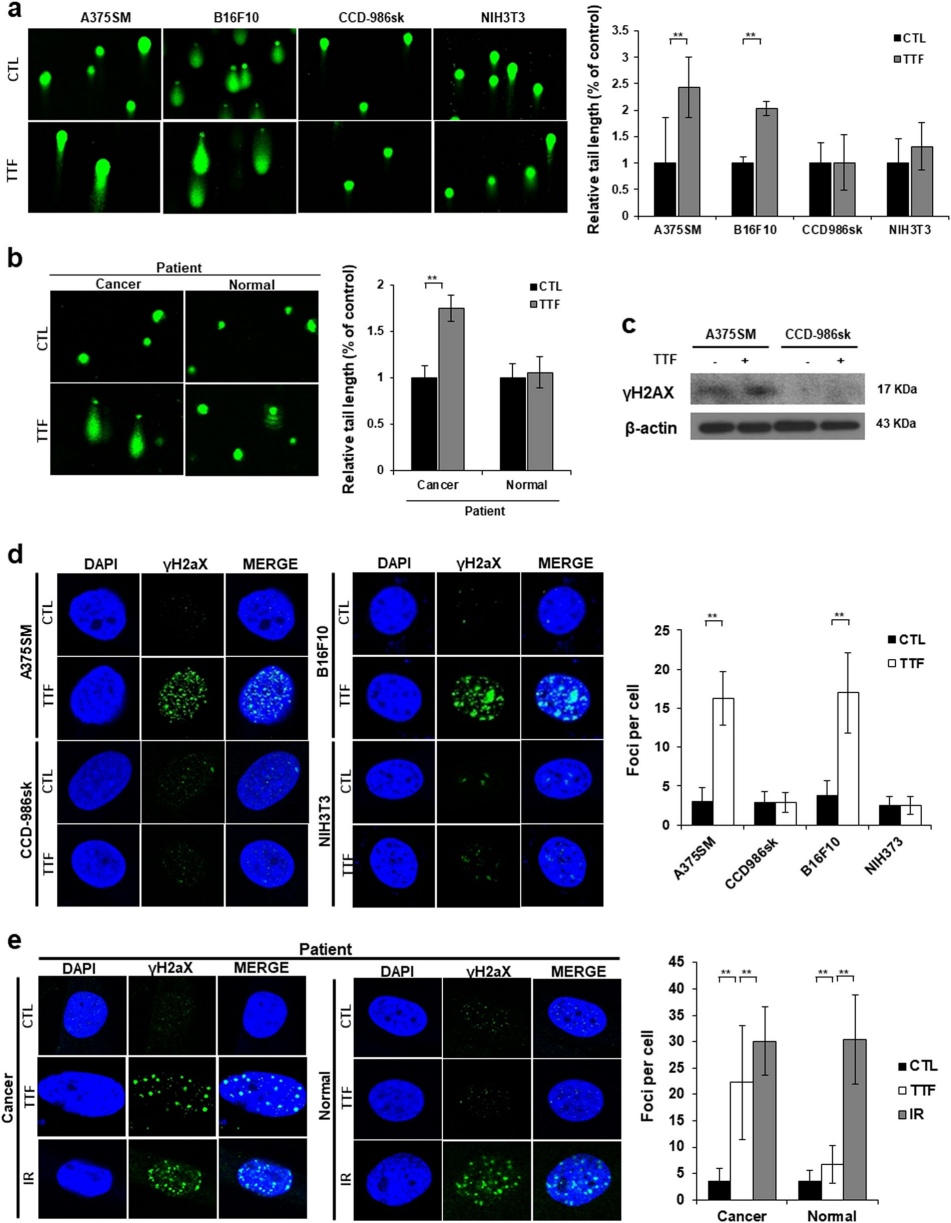
TTFs induce more DNA damage in tumor cells than that in normal cells. **a**, **b** Alkaline comet assay in cancer and normal cells treated with TTFs for 48 h. Quantitative analysis of tail movements. The values represent the means of three experiments ± SD. **c** Equal amounts of cell lysates (20 µg) were separated by electrophoresis and analyzed by western blotting using the indicated antibodies. **d**, **e** Immunocytochemistry of phosphorylated H2AX, which is a marker of the DNA damage response, in cancer and normal cells exposed to TTF. The values represent the means ± SD; **p < 0.01

To investigate whether TTFs induce damage to DNA in melanoma cells and normal cells, comet assays were performed, and an increase in the tail length was observed in the cancer cells compared with that in the normal cells (Fig. 2a, b). In the comet assay, the tail length is used as an indicator of DNA damage^14^. Thus, TTFs caused DNA damage in the cancer cells, but no significant damage was observed in the normal cells.

A method has been developed to visualize individual DSBs using an antibody against γH2AX, which is a phosphorylated derivative of histone H2AX found at the site of the initial DNA DSB^15^. Recently, TTF has been reported to induce γH2AX in GBM cancer cells^10^. Our results showed an increased expression of γH2AX only in cancer cells, and no significant difference was found in the normal cells (Fig. 2c). The same result was confirmed by the fluorescence microscopy observation (Fig. 2d, e).

### TTFs significantly reduce tumor volume within 9 days of treatment at 7 days after tumor establishment

**Fig. 3:**
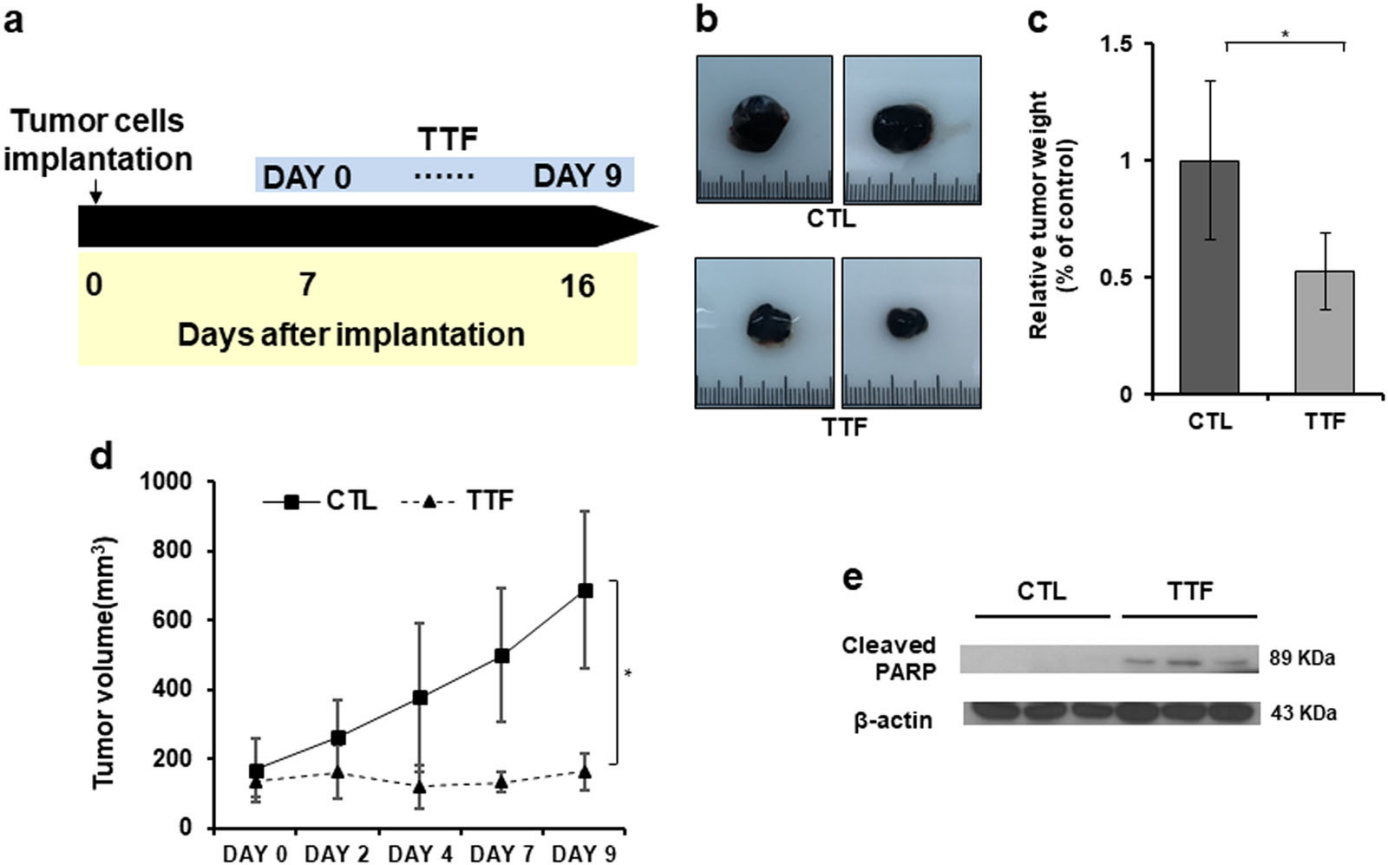
TTFs effectively inhibit tumor growth in vivo. **a** Schematic timeline of the in vivo experiments. **b** B16F10 melanoma cells were subcutaneously injected into nude mice. Tumor volumes were measured at the indicated time points. **c** Image of isolated tumors derived from control or TTF-treated mice. **d** Tumor weights at the time of killing. **e** Equal amounts of tumor tissue lysates (20 µg) were separated by electrophoresis and analyzed by western blotting using the indicated antibodies. The values represent the means ± SD; *p < 0.05

Prior to confirming the damage to the normal organs, we first assessed whether TTFs inhibited tumor growth in vivo. First, TTF treatment was performed at a 1 V/cm intensity for 9 days after subcutaneously injecting tumors into the backs of the mice (Fig. 3a). After the TTF treatment, the tumors in the control and treatment groups immediately separated. The tumor weight in the treatment group was approximately 50% of that in the control group, and the TTF treatment effectively suppressed tumor growth (Fig. 3b, c). The tumor volume measurements during the treatment also showed that the TTF treatment prevented the tumor from growing (Fig. 3d). Our results showed that the expression of cleaved PARP increased in the isolated tumor tissues in the treated group compared to that in the control group, indicating that TTFs induced apoptosis in vivo (Fig. 3e).

### TTF treatment for a long-term period does not cause any detectable pathologic abnormalities in normal tissues

**Fig. 4:**
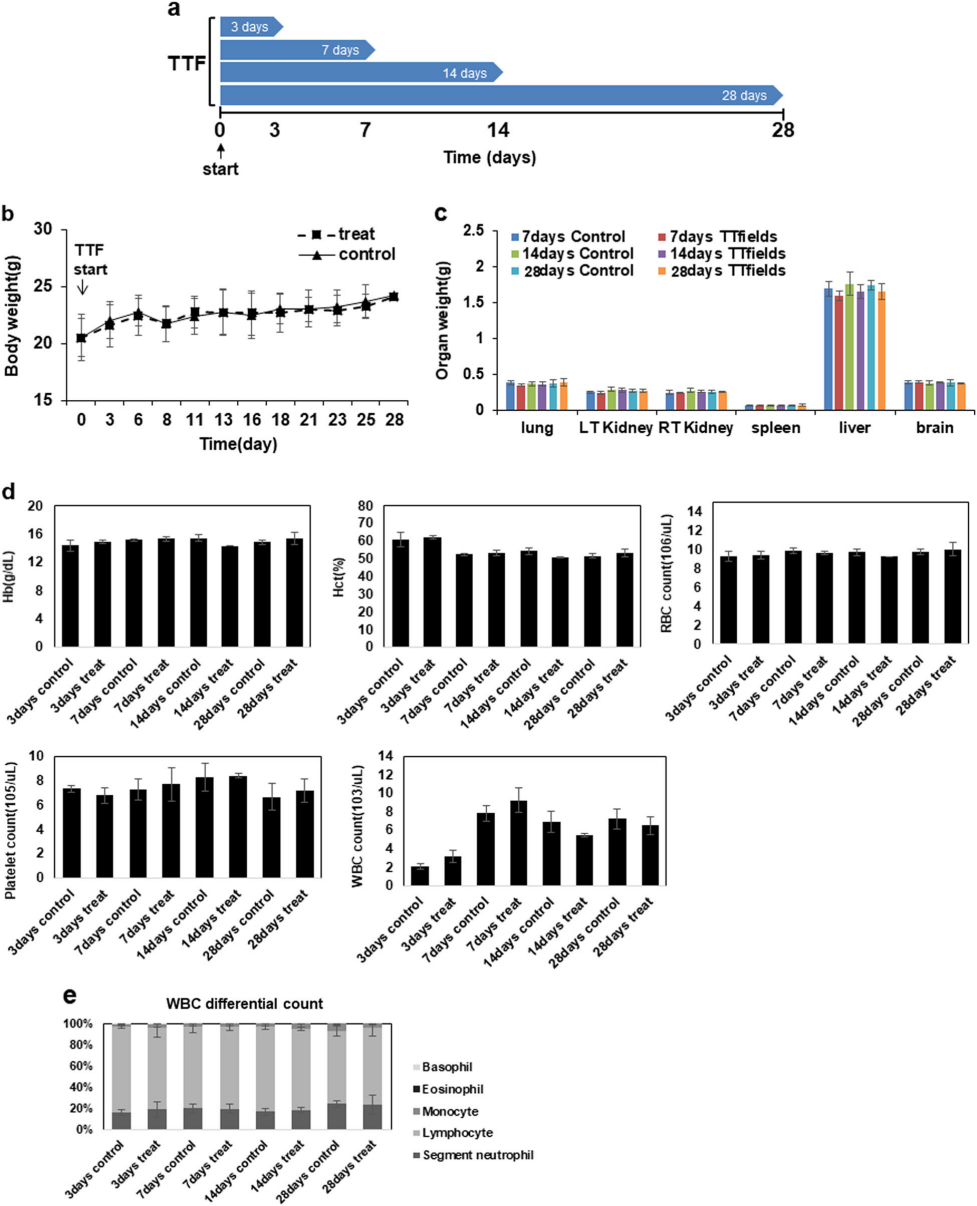

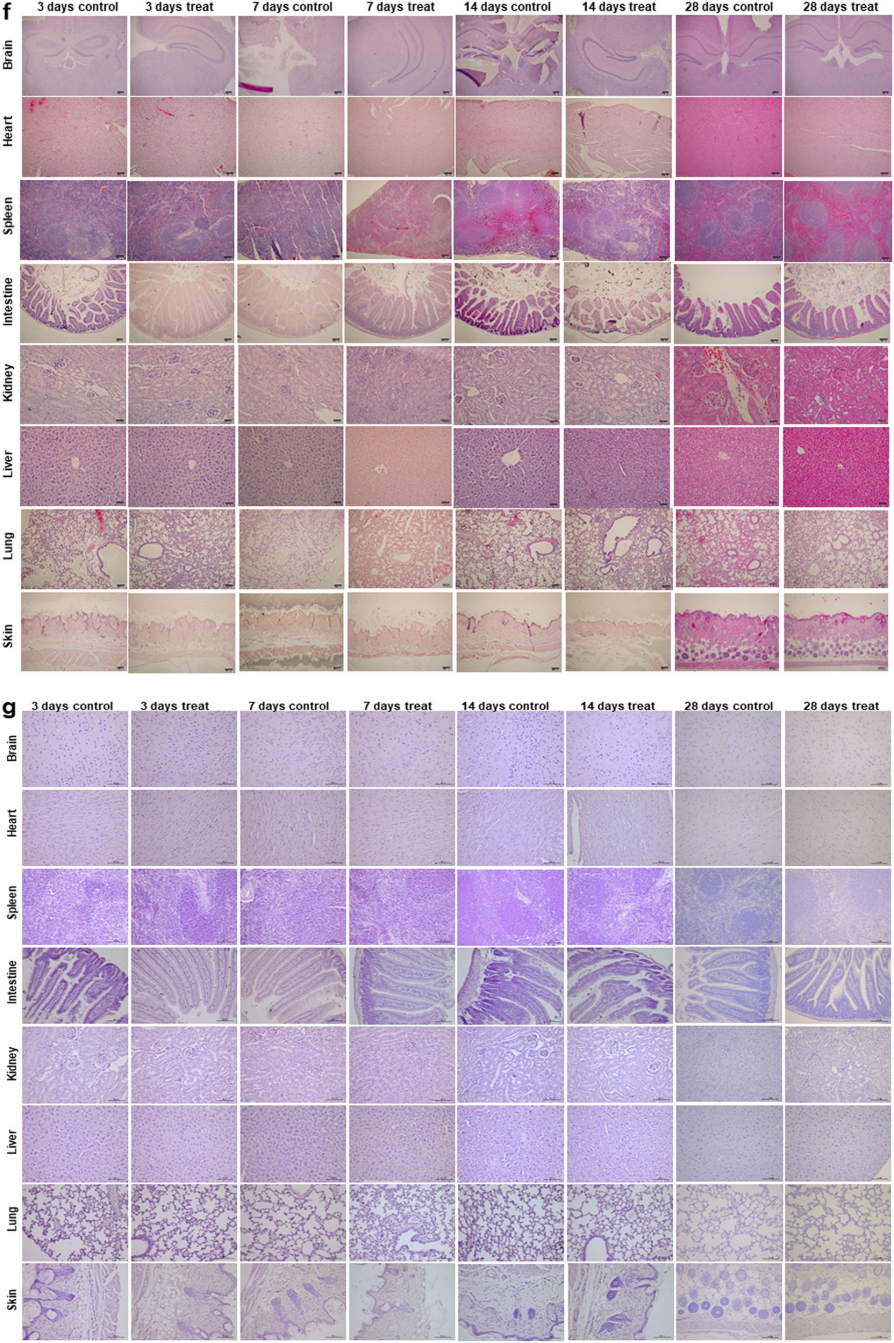
TTFs did not cause noticeable damage to normal tissue. **a** Schematic timeline of the in vivo experiments. **b** Body weight was measured in TTF-treated mice and nontreated mice. **c** Organ weights at the time of killing were measured. **d**, **e** CBC test results in blood samples from control and treated groups in vivo. **f** H&E staining was performed in 8 organs from the control and treated groups. **g** TUNEL assays were performed in 8 organs from the control and treated groups. The values represent the means ± SD

To examine normal tissue complications in vivo after prolonged TTF treatment, mice were treated for 3, 7, 14, and 28 consecutive days without injecting tumors (Fig. 4a). During the TTF treatment, the mice in the control and treatment groups exhibited negligible body weight differences, suggesting that the TTF treatment did not cause excessive stress in the treated mice (Fig. 4b). The differences in the weights of the organs between the control and treatment groups and the complete blood count (CBC) test results also did not reveal any noticeable differences (Fig. 4c–e). The H&E staining and TUNEL assays were performed using organs collected from the control mice and the mice treated for 3, 7, 14, and 28 days (Fig. 4f, g). The results also showed no abnormalities.

## Discussion

TTFs have been developed as a new modality for the treatment of cancer and have attracted increasing attention due to their excellent effect on intractable cancers, such as GBM. However, research investigating TTF-induced side effects in comparison with the excellent therapeutic effect of TTFs is lacking. Our research appears to be the first to show detailed results regarding the responses of cancer and normal cells based on both in vitro and in vivo experiments. The experimental results showed that in contrast to the significant damage to cancer cells, there is negligible TTF-induced apoptosis or DNA damage in normal cells. In the case of radiation therapy, which is similar to TTFs in terms of the usage of electromagnetic waves in treatment, the irradiated beam directly or indirectly ionizes atoms and consequently induces free radicals, resulting in DNA damage^16^. Radiation therapy eventually leads to the destruction and transformation of genes and apoptosis, even in normal cells. Normal cells, such as lymphocytes, spermatogonia, and serous cells in the salivary gland, have been reported to undergo apoptosis following radiation^17,18^, which could cause fatal side effects, such as fibrosis, heart disease, and the generation of secondary cancer due to radiation.

The lack of significant TTF-induced apoptosis or DNA damage may be due to the mechanism by which TTFs interact with cells. In contrast to radiation, TTFs appear to obstruct mitosis by preventing the alignment of the spindle and lead to cell cycle arrest and cell death^1,5^. Thus, nondividing cells or slowly dividing cells may not be affected by TTF application. In general, normal cells grow at a much slower rate than cancer cells, which probably explains why the normal cells were not significantly affected by the application of TTFs. In our experiment, the doubling times of CCD-986sk (doubling time: 40 h) and NIH3T3 (doubling time: 28 h) were much longer than those of A375SM (doubling time: 24 h) and B16F10 (doubling time: 20.1 h). Although the natural characteristics of a cell may affect how TTFs interfere with the cell, one of the most dominant factors appears to be the doubling time, and these results suggest that TTFs selectively act on fast growing cells but not slow growing cells.

In a randomized phase III trial involving recurrent glioblastoma patients, TTF and chemotherapy groups were compared. No significant difference was observed in the median survival, but the quality of life of the treated patients in the TTF group was superior to that of the patients in the chemotherapy group. The typical side effects of chemotherapy, such as anorexia, diarrhea, constipation, nausea, and vomiting, were not found in the patients treated with TTF, and only mild skin reactions were observed^7,19^. Tumor-targeting anticancer agents and radiation therapies, such as heavy ion therapy, have continued to evolve to maximize the effectiveness of tumor therapy with minimal damage to normal tissue. Therapeutic methods that target only malignant tumors without damaging normal tissues will be of great benefit to patients. TTFs can be a treatment method for malignant tumors. Since this treatment method is applied only to dividing cells by inhibiting the alignment of the spindle during mitosis, normal slowly dividing cells will be minimally damaged. In clinical trials, dermatitis has been reported to occur at the site of the electrode attachment^6^.

According to previous reports, 16% of patients treated with TTFs had irritated contact dermatitis (due to hydrogel, moisture, and alcohol) or allergic contact dermatitis in phase III trials^6^. In the worst cases, approximately 1% of patients had symptoms of ulcers and infections^7^. To prevent the dermatologic adverse events, suitable shaving to attach the arrays, the use of 70% isopropyl alcohol to better adhere the array to the skin, and exchanging the transducer array at least once every 3–4 days are recommended^6^. In the case of radiation therapy, skin reactions (radiation dermatitis) are common side effects of treatment and occur in approximately 90% of patients^20–22^. In our study, no skin reactions occurred in the mice treated with TTFs at 1 V/cm.

In addition, following radiotherapy, γH2AX expression is markedly increased in the brain, heart, small intestine, and lungs in mice that have been irradiated with 2 Gy in vivo. Thus, 2 Gy radiation, which is the clinical dose, induces DSBs and may damage normal organs^23^. In contrast, we confirmed that γH2AX expression did not increase following the application of TTF treatment to patient-derived primary cells under clinical intensity and frequency conditions in vitro, suggesting that this therapy is less likely to induce DSBs in normal tissues in patients who are treated with TTF.

In addition, approximately 30% of lung cancer patients who receive radiotherapy suffer from lung pneumonitis because there are particularly radio-sensitive tissues in the lung^24,25^. Unfortunately, there is no known cure to prevent acute or chronic radiation pneumonia, which is a side effect of this treatment^26^. Thus, the tolerance of doses in the lung is low, and the efficacy of radiotherapy is limited^27^. Significant lung damage has been reported in mice exposed to a fractionated dose of 30 Gy^25^. However, in TTF therapy, only 2% of patients experience respiratory disorders^10^, which is significantly lower than that for radiation therapy. No damage was observed in the lungs from mice that received TTF therapy in our study.

In conclusion, our study was the first to evaluate TTFs using patient-derived cells to ensure that TTFs have the least impact on normal tissues and the greatest impact on cancer tissues. We also assessed for the first time whether this included the death of normal cells by organ. This study is an indispensable contribution for expanding the clinical application of TTFs, and we confirmed that TTFs selectively act on cancer. This selectivity appears to be due to the difference in the doubling time between cancer cells and normal cells and is likely clinically meaningful because normal adult cells grow very slowly compared to cancer cells. However, our findings are limited to cell morphology and death, and we aim to proceed with further studies involving more detailed evaluations, including studies using normal cells that have a fast doubling time.

## Materials and methods

### Alternating electric field experimental setup

Very low-intensity (<3 V/cm), intermediate-frequency (100–300 kHz), alternating electric fields induced by insulated electrodes have been reported to inhibit the growth of various tumor cells and named TTFs. In this experiment, TTFs were generated with a pair of insulated wires connected to a function generator and a high-voltage amplifier that generated sine-wave signals ranging from 0 to 800 V. The applied electric field intensity and frequency were 1.2 V/cm and 150 kHz, respectively. We chose 1.2 V/cm as the field intensity because this intensity is very similar to that currently used in the clinic. For the irradiation, the cells were plated in 60 mm dishes and incubated at 37 °C under humidified conditions and 5% CO_2_ until reaching 70–80% confluence.

### Antibodies and chemicals

Anti-β-actin was purchased from Santa Cruz Biotechnology (Dallas, TX, USA). The anti-cleaved PARP1 and β-actin antibodies were obtained from Cell Signaling Technology (Danvers, MA, USA), and antiphosphorylated H2AX (γH2AX) was obtained from Millipore (Billerica, MA, USA).

### Cell culture

The patient-derived tissue was obtained with informed consent from a patient who underwent surgery at the Korea Institute of Radiological and Medical Sciences (Institutional Review Board No. K-1603–001–001), and a primary cell culture was established from this tissue. Briefly, the tissue was minced into a slurry with blades, washed with phosphate-buffered saline (PBS), and centrifuged for 3 min at 1000 rpm. Then, the supernatant was discarded, and the pellet was resuspended in serum-free Dulbecco’s modified Eagle’s medium (DMEM; WelGene, Daegu, Korea) containing 0.05–0.1% (w/v) collagenase type I (Gibco®, Life Technologies) to disaggregate the cells. After 2 h, the cells were washed thoroughly with PBS and maintained in DMEM with 20% (v/v) fetal bovine serum (FBS).

The A375SM cells were grown in Minimum Essential Medium (WelGene, Daegu, Korea) supplemented with 10% FBS, glutamine, HEPES, and antibiotics. The CCD-986sk cells were grown in Iscove's modified Dulbecco's medium (WelGene, Daegu, Korea) medium supplemented with 10% FBS, glutamine, HEPES, and antibiotics. The NIH3T3 and B16F10 cells were grown in DMEM (WelGene, Daegu, Korea) medium supplemented with 10% FBS, glutamine, HEPES, and antibiotics. The patient-derived primary cells were grown in DMEM medium supplemented with 20% FBS, glutamine, HEPES, and antibiotics. All cells were grown at 37 °C in a humidified incubator under 5% CO_2_.

### Detection of apoptotic cells through Annexin V staining

After TTF exposure for 48 h, the cells were immediately harvested. The cells were subsequently washed with ice-cold PBS, trypsinized, and resuspended in 1× binding buffer (10 mm HEPES/NaOH (pH 7.4), 140 mm NaCl, and 2.5 = mm CaCl2) at 1 × 10^6^ cells/mL. Aliquots (100 μL) of the cell solution were mixed with 5 μL of Annexin V-FITC (BioVision, Milpitas, CA) and 10 μL of PI stock solution (50 μg/mL in PBS) via gentle vortexing, followed by 15 min of incubation at room temperature in the dark. Buffer (400 μL, 1×) was added to each sample, which was then analyzed on a FACScan flow cytometer (Becton Dickinson, Franklin Lakes, NJ, USA). A minimum of 10,000 cells was counted in each sample, and the data analysis was performed using CellQuest software (BD Biosciences).

### Immunocytochemistry

Immunocytochemistry was performed to determine the nuclear distribution of γH2AX in individual cells. The cells were grown on chambered slides 1 day prior to the TTF treatment. The TTF treatment was performed for 48 h while the cells remained attached to the slides, followed by fixation with 4% paraformaldehyde and permeabilization with 0.5% Triton X-100 in PBS. The detection was performed after blocking the slides in 10% FBS/1% bovine serum albumin for 1 h with a 1:1000 dilution of a FITC-labeled mouse monoclonal antibody against γH2AX (Millipore, Billerica, MA, USA).

### Comet assay

To detect the single- and double-strand breaks, an alkaline comet (single-cell gel electrophoresis) assay was performed according to the manufacturer’s instructions (Enzo Life Sciences, Farmingdale, NY). The cells were plated in 100 mm tissue culture dishes at 1 × 10^6^ cells/dish and incubated overnight. After the TTF exposure for 48 h, the cells were immediately harvested. The cells were lysed at 4 °C for 1 h in lysis buffer and subjected to alkailne electrophoresis buffer at 4 °C. To detect the DNA, the slides were stained with ethidium bromide and examined for fluorescence emission at a 515–560 nm excitation filter and a 590 nm barrier filter. The DNA damage was quantified through the open comet imageJ plugin to integrate the fluorescence intensity.

### Western blotting

After TTF treatment for 48 h, the cells were lysed with RIPA buffer, and the proteins were separated via sodium-polyacrylamide gel electrophoresis and transferred to nitrocellulose membranes. The membranes were blocked with 1% (v/v) nonfat dried milk in Tris-buffered saline with 0.05% Tween 20 and incubated with the required antibodies. The primary antibodies were used at a 1:1000 dilution (5% bovine serum albumin) and secondary antibodies at a 1:5000 dilution (5% skim milk). The immunoreactive protein bands were visualized via enhanced chemiluminescence (Amersham Biosciences) and scanned.

### Tumor xenografts in nude mice

A single-cell suspension (2 × 10^4^ cells) was subcutaneously injected into the flanks of 5-week-old NCR nude mice (Nara Biotech.). When the tumor reached a minimal volume of 100 to 200 mm^3^, TTF treatment was started. Tumor volumes were determined according to the formula (*L* × *l*^2^)/2 by measuring tumor length (*L*) and width (*l*) with calipers. The animal protocol was approved by Korea University Institutional Animal Care and Use Committee (IACUC) (KUIACUC-2017–153).

### H&E staining

The mice in the control and treatment groups were treated for 3, 7, 14, and 28 days. For the histopathological assessment, fixed organs and tumors were embedded in paraffin blocks, followed by cutting into 4 μm sections and mounting on glass slides for the H&E staining.

### TUNEL assay

The mice in the control and treatment groups were treated for 3, 7, 14, and 28 days and used for the in vivo apoptosis studies. The spleens, hearts, lungs, livers, skins, brains, and intestines were collected and fixed with 10% neutral-buffered formalin. Deparaffinized sections were incubated with 20 μg/mL protease K for 15 min at room temperature, washed with PBS, and incubated with TUNEL reaction mixture (Millipore, Burlington, MA, USA) for 1 h at 37 °C in a humidified chamber. Then, the tissue sections were incubated with an anti-digoxigenin-peroxidase mixture and subsequently the peroxidase substrate. The images were acquired using a Nikon Eclipse Ts2R-FL.

### Statistical analysis

Statistical significance was determined by Student’s *t*-test. Differences were considered significant if the *p* value was less than 0.05 or 0.01.

## Electronic supplementary material

Supplementary Figure

Supplementary legends
