# Supplementary figures and images for: Selective toxicity of tumor treating fields to melanoma: an in vitro and in vivo study
Source: Cell Death Discov. 2018 Oct 3;4:46. doi: 10.1038/s41420-018-0106-x (PMC6170382; doi:10.1038/s41420-018-0106-x)

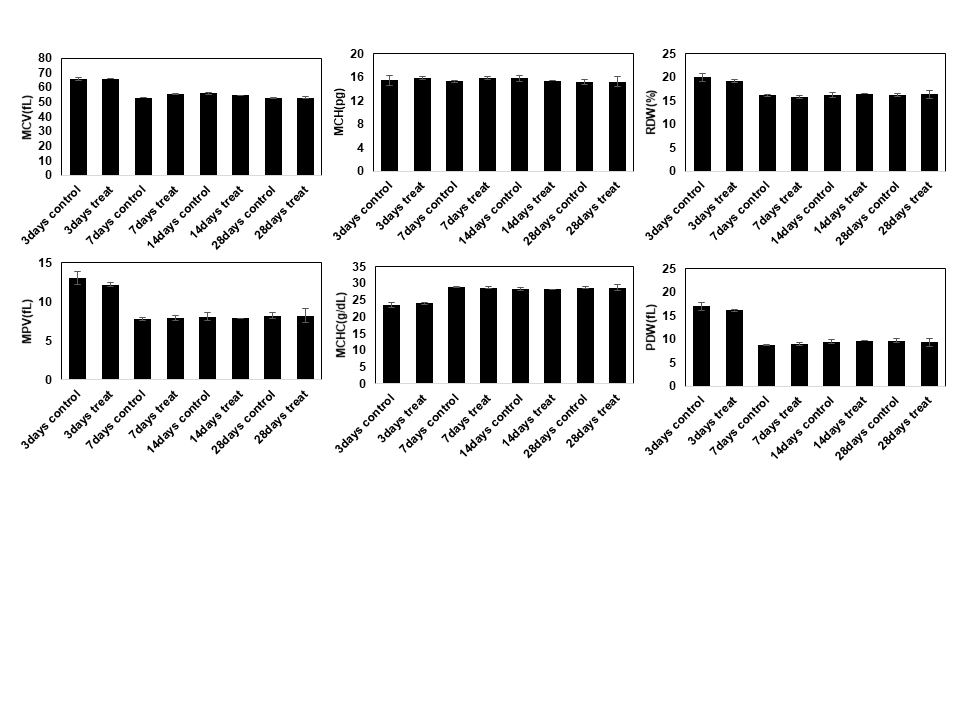

Supplement: Supplementary file 1 — Supplementary Figure [file 41420_2018_106_MOESM1_ESM.tif]
