# Supplementary material for: Selective toxicity of tumor treating fields to melanoma: an in vitro and in vivo study
Source: Cell Death Discov. 2018 Oct 3;4:46. doi: 10.1038/s41420-018-0106-x (PMC6170382; doi:10.1038/s41420-018-0106-x)
Supplement: Supplementary file 2 — Supplementary legends [file 41420_2018_106_MOESM2_ESM.docx]

**FIGURE LEGENDS**

**Supplementary figure 1.** CBC test results in blood samples from control and treated groups *in vivo*.
